# Supplementary material for: Impact on All-Cause and Cardiovascular Mortality Rates of Coronary Artery Calcifications Detected during Organized, Low-Dose, Computed-Tomography Screening for Lung Cancer: Systematic Literature Review and Meta-Analysis
Source: Cancers (Basel). 2021 Mar 28;13(7):1553. doi: 10.3390/cancers13071553 (PMC8036563; doi:10.3390/cancers13071553)
Supplement: Supplementary file 1 [file cancers-13-01553-s001.pdf]

Supplementary Materials: Impact on All-Cause and Cardiovascular Mortality Rates of Coronary Artery Calcifications Detected during Organized, Low-Dose, Computed-Tomography Screening for Lung Cancer: Systematic Literature Review and Meta-Analysis

Sébastien Gendarme, Helene Goussault, Jean-Baptiste Assié, Cherifa Taleb, Christos Chouaïd and Thierry Landre

Table S1. Eligibility criteria for study inclusion in the meta-analysis.

	Inclusion Criteria	Exclusion Criteria
Population	Patients > 18 years old, active or ex-smokers included in an, organized program of low-dose thoracic computed-tomography (CT) scanning to screen for lung cancer	Children<br>Screening of groups at risk for lung cancer because of exposure(s) other than tobacco<br>Subjects with symptoms of lung cancer<br>Subjects diagnoses with lung cancer
Interventions	Low-dose CT scan within an organized program to screen for lung cancer	Thoracic CT scan not obtained during a screening program<br>Stop-smoking program without CT scan
Judgement criterion	Relative risk/hazard ratio/odds ratio	Cost/efficacy<br>Qualitative study
Type of study	Randomized–controlled trial, meta-analyses, cohort, case–control or series of cases published after 2001	Case reports, editorial, expert opinion, no control group
Study duration	No prerequisite	None

Table S2. Grid to evaluate the quality study ("Checklist for the assessment of the methodological quality", Downs and Black 1998 [17]) of the six studies included in the meta-analysis.

Quality Standards	Jacobs 2012 [20]	Lessmann 2019 [21]	Sverzellati 2012 [22]	Shemesh 2010 [25]	Rasmussen 2015 [24]	Puliti 2019 [23]
Study Quality						
1. Is the hypothesis/aim/objective of the study clearly described?	Yes	Yes	Yes	Yes	Yes	Yes
2. Are the main outcomes to be measured clearly described in the Introduction or Methods section?	Yes	Yes	Yes	Yes	Yes	Yes
3. Are the characteristics of the patients included in the study clearly described?	Yes	Yes	Yes	Yes	Yes	Yes
4. Are the interventions of interest clearly described?	Yes	Yes	Yes	Yes	Yes	Yes
5. Are the distributions of principal confounders in each group of subjects to be compared clearly described?	Yes	Yes	Yes	Yes	Yes	No
6. Are the main findings of the study clearly described?	Yes	Yes	Yes	Yes	Yes	Yes
7. Does the study provide estimates of the random variability in the data for the main outcomes?	Yes	Yes	Yes	Yes	Yes	No
8. Have all important adverse events that may be a consequence of the intervention been reported?	Not described	Not described	Not described	Not described	Not described	Not described
9. Have the characteristics of patients lost to follow-up been described?	Yes	Yes	No	No	Yes	No
10. Have actual probability values been reported (e.g. 0.035 rather than <0.05) for the main outcomes except where the probability value is less than 0.001?	No	Yes	Yes	Yes	Yes	Yes
External Validity						
11. Were the subjects asked to participate in the study representative of the entire population from which they were recruited?	Yes	Yes	Yes	Yes	Yes	Yes
12. Were those subjects who were prepared to participate representative of the entire population from which they were recruited?	Yes	Yes	Yes	Yes	Yes	Yes
13. Were the staff, places, and facilities where the patients were treated, representative of the treatment the majority of patients receive?	Yes	Yes	Yes	Yes	Yes	Yes
Internal Validity – Bias						
14. Was an attempt made to blind study subjects to the intervention they have received?	No	No	No	No	No	No
15. Was an attempt made to blind those measuring the main outcomes of the intervention?	Yes	Not described	Yes	No	Not described	Not described

16. If any of the results of the study were based on "data dredging", was this made clear?	Not involved	Not involved	Not involved	Not involved	Not involved	Not involved
17. In trials and cohort studies, do the analyses adjust for different lengths of follow-up of patients, or in case-control studies, is the time period between the intervention and outcome the same for cases and controls?	Yes	Yes	Yes	No	Yes	Yes
18. Were the statistical tests used to assess the main outcomes appropriate?	Yes	Yes	Yes	Yes	Yes	Yes
19. Was compliance with the intervention/s reliable?	Yes	Yes	Yes	Yes	Yes	Yes
20. Were the main outcome measures used accurate (valid and reliable)?	Yes	Yes	Yes	Yes	Yes	Yes
Internal Validity - Confounding (Selection Bias)						
21. Were the patients in different intervention groups (trials and cohort studies) or were the cases and controls (case-control studies) recruited from the same population?	Yes	Yes	Yes	Yes	Yes	Yes
22. Were study subjects in different intervention groups (trials and cohort studies) or were the cases and controls (case-control studies) recruited over the same period of time?	Yes	Yes	Yes	Yes	Yes	Yes
23. Were study subjects randomised to interventiongroups?	Yes	Yes	Yes	No	Yes	Yes
24. Was the randomised intervention assignment concealed from both patients and health care staff until recruitment was complete and irrevocable?	Yes	Yes	Yes	Not involved	Yes	Yes
25. Was there adequate adjustment for confounding in the analyses from which the main findings were drawn?	Yes	Yes	Yes	No (HTA not considered)	Yes	No (HTA not considered)
26. Were losses of patients to follow-up taken into account?	No	No	No	No	No	No
27. Did the study have sufficient power to detect a clinically important effect where the probability value for a difference being due to chance is less than 5%?	Yes	Yes	Yes	Yes	Yes	Yes

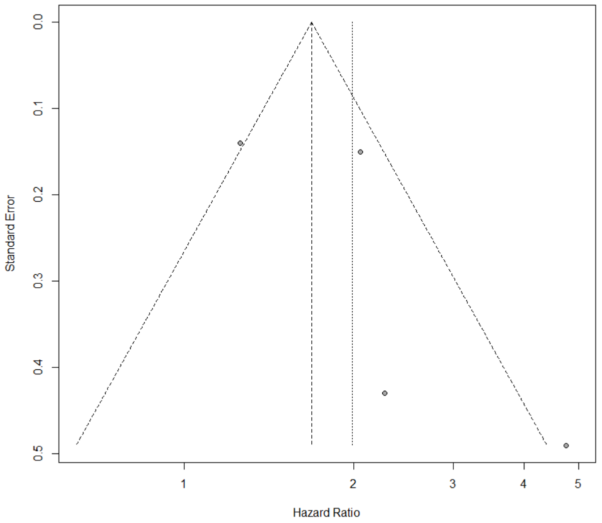

Funnel plot (meta-analysis 1) of CV mortality (CAC > 0)

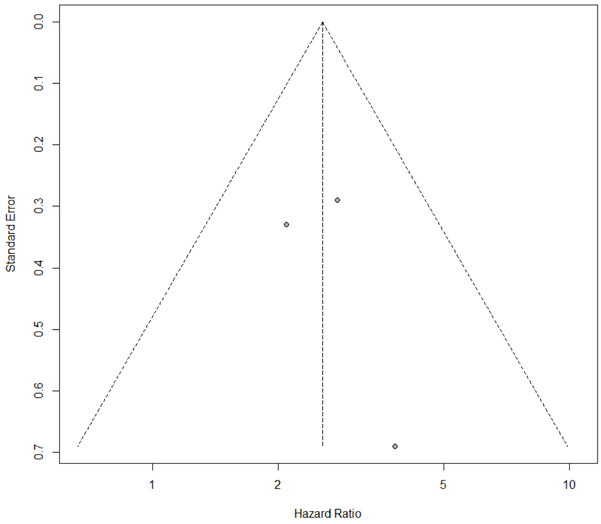

Funnel plot (meta-analysis 2) CV mortality (high CAC score)

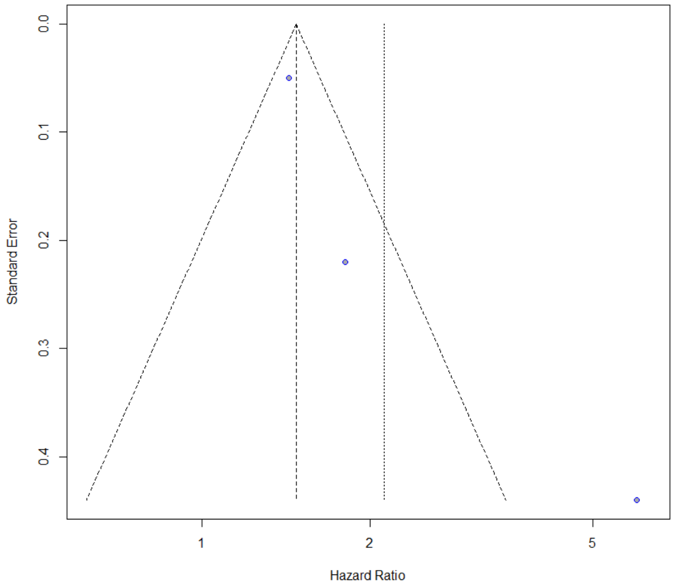

Funnel plot (meta-analysis 3) all-cause mortality (CAC > 0)

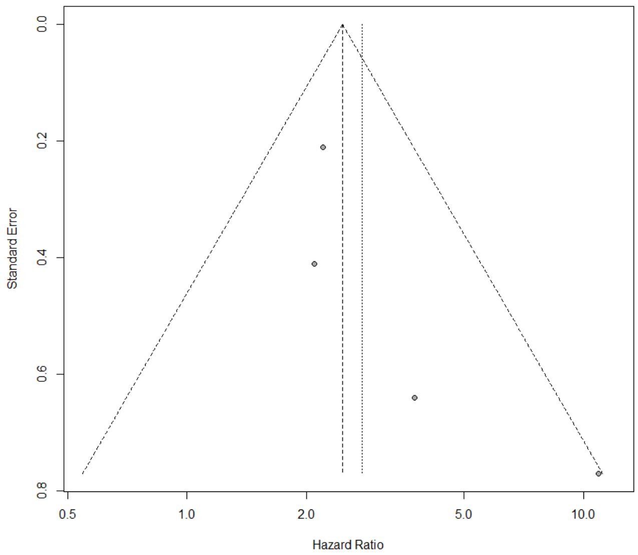

Funnel plot (meta-analysis 4) all-cause mortality (high CAC score)

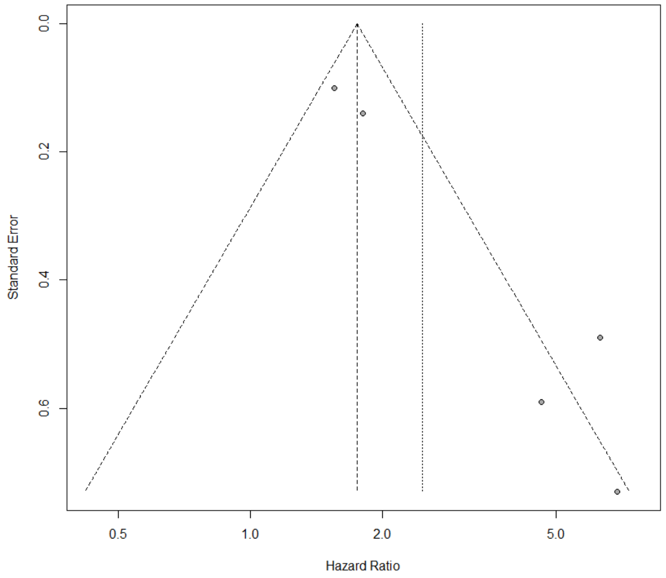

Funnel plot (meta-analysis 5) CV events and mortality as a function of sex

Figure S1. Study of publication biais**.**
